# Comparing three screen-based sedentary behaviours’ effect upon adolescents’ participation in physical activity: The ESSENS study

**DOI:** 10.1371/journal.pone.0241887

**Published:** 2020-11-25

**Authors:** Arthur Chortatos, Sigrun Henjum, Liv Elin Torheim, Laura Terragni, Mekdes K. Gebremariam

**Affiliations:** 1 Department of Nursing and Health Promotion, Faculty of Health Sciences, OsloMet–Oslo Metropolitan University, Oslo, Norway; 2 Department of Nutrition, Institute of Basic Medical Sciences, University of Oslo, Oslo, Norway; The Education University of Hong Kong, HONG KONG

## Abstract

**Background:**

Literature focusing on the association between sedentary behaviours and physical activity has provided equivocal results and has been dominated by TV viewing as the indicator of sedentary behaviour. There is a need for more studies exploring the association between contemporary screen activities and physical activity among youth.

**Methods:**

A cross-sectional study including 742 adolescents was conducted in 2016. Data were collected at school through an online questionnaire. Regression analyses were used to explore the association between different screen-based sedentary behaviours and participation in physical activity.

**Results:**

The results showed that those with lower (vs higher) time spent on TV/movie streaming and electronic game playing both on weekdays and weekend days had significantly higher odds of participating in physical activity. There were no significant associations between socializing/surfing online both on weekdays and weekend days and physical activity in adjusted models.

**Conclusions:**

TV/movie streaming and electronic game playing during both weekdays and weekend days were significantly inversely related with participating in physical activity. Initiatives aimed at reducing screen-based sedentary activities might result in favourable effects on physical activity levels among adolescents.

## Background

Sedentary behaviour (SB) amongst adolescents has increasingly been recognized as an important behaviour with negative health consequences, and research in the area has grown significantly in recent decades. SBs have been defined as any waking behaviour characterized by an energy expenditure ≤1.5 metabolic equivalents (METs), so that someone sitting, reclining, or lying down would be considered as being engaged in SB [1]. Common SBs include TV viewing, electronic gaming, socializing or browsing (surfing) the internet (collectively termed “screen time”), as well as sitting in automobiles and reading [1, 2].

Contemporary adolescents are offered an abundance of channels encouraging SB, the majority associated with use of electronic devices such as television, computers, mobile phones, and gaming consoles, activities collectively designated as screen-based SBs [3].

There are several health concerns for adolescents which are attributed to SB, including an increased risk of weight gain and obesity [4–6], an increased risk of metabolic syndrome [7], as well as cardiovascular disease, diabetes, and depression [8]. Yet different forms of SB have been reported as having dissimilar relationships with some non-communicable diseases. One study reported electronic gaming as having no association with overweight amongst 16 year old adolescents, even though television viewing amongst the same sample did [9]. Although high levels of SB combined with minimal levels of physical activity (PA) are frequently cited as being involved in the aetiology of non-communicable diseases [10], this view may not be as straightforward as it seems. The so-called ‘displacement theory’ hypothesizes that time spent in SBs displaces time that could have been spent being physically active [11]. The reported association between screen-based SB and reduced PA is somewhat conflicting [12]. While an inverse association between PA and screen time has previously been reported [13, 14], others have observed no significant association [15, 16]. The fact that much of the literature has focused upon TV viewing as the most prevalent indicator of SB may be, to some extent, outdated when the present ubiquity of available electronic devices is considered. As types of technologies promoting SB have flourished, new approaches in researching the phenomenon are required, with newer screen-based technologies dominating, making television alone an unreliable marker of adolescent SB [17, 18]. Furthermore, there have been different associations with PA reported for different screen-based SBs [3, 19]. There is therefore a need for more studies incorporating SBs other than TV viewing, in particular more contemporary SBs. In addition, time available for PA and SB is somewhat different during weekdays compared to weekend days for most of the population, in particular adolescents [20, 21]. It could thus be hypothesized that “displacement” is more likely to occur during weekend days compared to weekdays, when there might be more leisure time available for either screen-based SB or PA. Conducting separate analyses for weekdays and weekend days would help test this hypothesis.

Against this background, this study aimed to assess whether time spent performing three different screen-based SBs (television/movie streaming, electronic gaming, and online surfing or socializing) by adolescents was associated with time spent on PA. Potential differences between weekdays and weekend days were also explored.

## Methods

### Design and sample

The participants in this study were students from secondary schools participating in the Environmental determinantS of dietary BehaviorS among adolescENtS (ESSENS) cross-sectional study in the Øvre Romerike district of South East Norway. All secondary schools in the region were included. Of the twelve secondary schools invited to participate in the study, eleven accepted the invitation. All eighth graders in the included schools were then invited to participate in the study. Participants were invited using invitation letters distributed by teachers at school, containing information about the project for both parents and adolescents, as well as parental consent forms.

In total, 1,163 adolescents in the eighth grade (age 13–14 years) were invited to participate in this study and a total of 781 (67%) received parental consent for participation. In total, 742 adolescents (64% of those invited and 95% of those with parental consent) participated in the study which was conducted at schools between October and December 2016. The study was approved by the Norwegian Centre for Research Data. Written informed consent was obtained from all parents of participating adolescents who themselves provided assent.

### Data collection

A web-based questionnaire was used to collect data from the adolescents, using the LimeSurvey data collection tool. The questionnaires were answered at school, taking approximately 30–45 min to complete, and queried respondents about different lifestyle behaviours and their determinants including PA and SB. Research group members were present during data collection to answer questions and make sure the adolescents responded independently from each other. The questionnaire was pre-tested for clarity and length among a group of adolescents of the same age as the study participants (n = 23), prior to the main study.

### Measures

#### Sociodemographic measures

Two questions assessing parental education were included on the parental informed consent form for the adolescent. Parental education was categorised as low (12 years or less of education, which corresponded to secondary education or lower) or high (13 years or more of education, which corresponded to university or college attendance). Educational level of the parent with longest education, or else the one available, was used in the analyses. Participants were divided into either ethnic Norwegian or ethnic minority, with minorities defined as those having both parents born in a country other than Norway [22].

#### Sedentary behaviour and physical activity

Three types of screen-based SB were included: television/movie streaming, electronic gaming, and online surfing or socializing. One separate question was used to assess each SB asking participants to report how much time on average they had spent on such activities (e.g. ‘How many hours do you usually watch TV as well as DVD, video or films on a PC, TV or iPad…play computer games or electronic games (Playstation, XBOX etc), or play on an iPad/tablet or mobile phone…use a computer, iPad/tablet or mobile phone for activities such as chatting, e-mails, internet surfing, Facebook or Instagram etc. in you free time during weekdays’), with separate questions asked for weekdays and weekends. A response scale ranging from ‘none’ to ‘>4 hours’ spaced by half hour increments were available as options. These questions have shown evidence of test-retest reliability among 10–12 year old European children [23].

All SBs were sorted into three groups based on the relationship to the median time spent on such activities (all values up to and including the median, 30 minutes to 1 hour above the median, and all values >1 hour above the median), as the distribution for all screen-based SB activities was skewed. Our decision to further divide the groups above the median into ‘above’ and ‘well above’ the median was made in order to reveal characteristics that may exist between frequent and excessive screen-based SB individuals, whilst keeping sample distribution robust between the three groups. As the three SBs in either weekdays or weekend days have differing medians, the actual number of hours for the three groups are outlined in each screen-based SB analyses presented in the results.

Frequency of participation in PA was assessed from one question asking participants to choose how many days in an average week they usually participated in at least 60 minutes of PA (from a scale of 0 to 7 days). The responses were further dichotomised to those participating in PA for at least 60 minutes <5 days per week or else ≥5 days per week. Evidence of good test-retest reliability and good correlations of the measure with objectively measured PA have previously been documented [24, 25].

### Confounders

The following confounders were adjusted for in the regression analyses: the presence of bedroom TV, access to electronic equipment, non-screen-based SBs, as well as self-efficacy for use of screen-based equipment and performing PA.

#### Non-screen-based sedentary behaviour

Time spent on non-screen-based SB was assessed using two questions [‘How many hours do you use for homework or reading a book?’, and ‘How many hours do you use doing hobbies, handcrafts or music practise?’], with possible responses ranging from ‘<30 minutes’ to ‘>3 hours’. These questions have shown evidence of good test–retest reliability among 11–15 year old Australian children [26].

#### Self-efficacy measures

Self-efficacy for control over television/movie streaming, as well as electronic gaming, was assessed using five questions [e.g. How sure are you that you can….limit television/movie streaming and computer gaming to at least 1 hour on at least one school day], with responses of either certain or uncertain available. Three of the 5 questions focussed upon school days whilst 2 focussed upon days of the weekend. Those having responded ‘certain’ to three or more of the five questions was designated as having a high self-efficacy for television viewing and/or gaming.

Self-efficacy for PA was assessed using a Likert-type scale with 5 questions [e.g. How much do you agree or disagree with the following…I am able to be physically active most days] with answer categories ranging from completely disagree to completely agree. Results from the five questions were combined and divided by five for an aggregate score ranging from 1 (very high) to 5 (very low). Responses were further categorised as those with ‘high’ self-efficacy (score of 2 or less, from a scale of 1–5) or ‘low’ self-efficacy (over 2, from a scale of 1–5). See S1 File for details of questions used.

### Statistical analyses

The study sample was firstly organized by three distinct screen-based SBs (TV/movie streaming, electronic gaming, and surfing/socializing online), with each screen-based SB sample divided into three median-based groups (those engaged in screen-based SB up to and including the median for the sample (low use), those engaged in screen-based SB 30 minutes to 1 hour above the median (medium use), and those engaged in screen-based SB >1 hour above the median (high use). Separate analyses were conducted for weekday and weekend assessments. Results are presented as frequencies (%), with chi-square tests performed to examine differences in sociodemographic and behavioural characteristics between the three groups.

Further logistic regression analyses were performed to assess the adjusted associations between screen-based SB and the reported frequency of participating in PA for at least 60 minutes ≥5 days per week, both for weekday and weekend screen-based SB. Due to their associations with the screen-based SBs in the sample, the following were controlled for in the analyses: gender, ethnicity, parental education, self-efficacy for television watching, self-efficacy for PA, having a television in bedroom, owning own PC, number of PC’s in the home, doing homework (during the weekdays for weekday analyses, and during the weekend for weekend analyses), and doing hobbies (during the weekdays for weekday analyses, and during the weekend for weekend analyses). In addition to these, logistic regression analyses regarding electronic gaming were also controlled for self-efficacy for electronic games. Results are presented as crude odds ratios (cOR) and adjusted odds ratios (aOR) with 95% confidence intervals (95% CIs). Collinearity between the predictors was investigated by calculating the variance inflation factor (VIF). The risk for collinearity was considered low if all the variables had a VIF value below two, as was the case in the regression models used, suggesting no collinearity existed within the predictors. Because schools were the unit of measurement in this study, we checked for clustering effect through the linear mixed model procedure. Due to a negligible clustering effect, multilevel analyses were not conducted.

A significance level of 0.05 was used. All analyses were performed using SPSS 25.0 (IBM Corp, Armonk, NY, USA).

## Results

### Sample demographics, sedentary behaviour and physical activity characteristics

The mean age of the survey sample was 13.6 years ± 0.3 standard deviation, with 54% of the 742 participants being females, and 91% ethnic Norwegian (Table 1). Seventy-seven percent of the sample reported doing ≥1 hour of homework per day during the weekdays, although on weekend days this contracted to 36%. Conversely, the number practicing hobbies during the weekdays and weekend ≥1 hour remained similar (56% weekdays, 53% weekends). Almost half the sample (47%) participated in over 60 minutes PA for ≥5 days in the average week.

**Table 1:** Socio-demographic characteristics, screen-based sedentary behaviour, non-screen-based sedentary behaviour, and physical activity patterns for entire sample (n = 742).

	n (%)
Gender (female)	395 (53.2)
Parental education^a^ (≥13y)	422 (59.8)
Ethnicity^a^ (minority)	68 (9.2)
Adolescents watching TV during weekdays^a^ (≥3 hours/day)	173 (24.0)
Adolescents watching TV during weekend days^a^ (≥4 hours/day)	120 (16.6)
Adolescents playing electronic games during weekdays^a^ (≥2.5 hours/day)	163 (22.6)
Adolescents playing electronic games during weekend days^a^ (≥3 hours/day)	195 (27.2)
Adolescents socializing/surfing online during weekdays^a^ (≥3 hours/day)	171 (23.7)
Adolescents socializing/surfing online during weekend days^a^ (≥3 hours/day)	207 (28.7)
Doing homework during weekdays^a^ (≥1 hour/day)	544 (77.0)
Doing homework during weekend days^a^ (≥1 hour/day)	249 (35.8)
Doing hobbies during weekdays^a^ (≥1 hour/day)	395 (55.9)
Doing hobbies during weekend days^a^ (≥1 hour/day)	364 (52.6)
Number of days >60 minutes PA in average week^a^ (≥5 days)	336 (47.1)

^a^Minor discrepancies in size from sample total exist owing to missing values.

### Television viewing habits

#### Weekday

The median time spent watching TV by the adolescents on weekdays was 1.5 hours. A total of 53% of adolescents watched TV ≤1.5 hours (low use group), 24% between 2–2.5 hours (medium use group), and 24% ≥3 hours per weekday (high use group). The highest proportion of those with highest parental education was found amongst the low use group (Table 2). There was a significant association between PA and TV viewing during weekdays (p<0.001). The percentage of those reporting PA over 60 minutes ≥5 days was 52.6%, 50.9%, 31.0% among the low use, medium use and high use groups respectively.

**Table 2:** Socio-demographic and physical activity (PA) characteristics for sample total (n = 742), grouped by low, medium and high TV use for weekday and weekend.

**TV use**	Low use^b^	Medium use	High use	P value^c^
**Weekday**	n (%)	n (%)	n (%)	
n = 722^a^	379 (52.5)	170 (23.5)	173 (24.0)	
Gender (female)	208 (54.9)	88 (51.8)	91 (52.6)	0.76
Parental education (≥13y)	237 (65.7)	98 (60.1)	81 (48.5)	0.001
Ethnicity (minority)	38 (10.0)	18 (10.6)	9 (5.2)	0.13
Number of days >60 minutes PA in average week				
≥5 days	195 (52.6)	85 (50.9)	53 (31.0)	<0.001
**Weekend**	Low use^d^	Medium use	High use	
n = 722^a^	422 (58.5)	180 (24.9)	120 (16.6)	
Gender (female)	229 (54.3)	99 (55.0)	59 (49.2)	0.56
Parental education (≥13y)	253 (63.2)	103 (59.2)	60 (51.3)	0.06
Ethnicity (minority)	37 (8.8)	18 (10.0)	10 (8.3)	0.86
Number of days >60 minutes PA in average week				
≥5 days	208 (50.4)	91 (51.4)	34 (31.4)	<0.001

^a^Minor discrepancies in size from sample total exist owing to missing values.

^b^Low use: adolescents watching TV during weekday for 0–1.5 hours; Medium use: 2–2.5 hours; High use: adolescents watching ≥3 hours (per day during weekdays).

^c^Chi-square test between groups categorized by hours of TV viewing during the weekday or weekend.

^d^Low use: adolescents watching TV during weekend 0–2.5 hours; Medium use: 3–3.5 hours; High use: adolescents watching ≥4 hours (per day during weekend days).

#### Weekend

The median time spent watching TV by the adolescents on weekend days was 2.5 hours. A total of 58% of adolescents watched TV ≤2.5 hours, 25% between 3–3.5 hours, and 17% ≥4 hours per weekend. Weekend analyses presented no significant differences regarding socio-economic characteristics for the sample grouped by median-based categories (Table 2). There was a significant association between PA and TV viewing during weekend days (p<0.001). The percentage of those reporting PA over 60 minutes ≥5 days was 50.4%, 51.4%, 31.4% among the low use, medium use, and high use groups respectively.

### Electronic gaming habits

#### Weekday

The median time spent playing online games by the adolescents on weekdays was 1 hour. A total of 60% of adolescents played electronic games ≤1hour, 17% between 1.5–2 hours, and 23% ≥2.5 hours per weekday. When comparing socio-demographic characteristics of the sample grouped by median-based categories for weekday gaming, significant associations were identified for gender and parent education, where significantly higher proportions of females and those with parents educated ≥13 years were found amongst the low use group (Table 3). There was a significant association between PA and electronic game playing during weekdays (p<0.001). The percentage of those reporting PA over 60 minutes ≥5 days was 51.4%, 50.0%, 33.1% among the low use, medium use and high use groups respectively.

**Table 3:** Socio-demographic and physical activity (PA) characteristics for sample total (n = 742), grouped by low, medium and high electronic game playing for weekday and weekend.

**Electronic gaming**	Low use^b^	Medium use	High use	P value^c^
**Weekday**	n (%)	n (%)	n (%)	
n = 721^a^	434 (60.2)	124 (17.2)	163 (22.6)	
Gender (female)	303 (69.8)	38 (30.6)	46 (28.2)	<0.001
Parental education (≥13y)	268 (64.3)	75 (63.0)	74 (47.7)	0.001
Ethnicity (minority)	38 (8.8)	7 (5.6)	19 (11.7)	0.21
Number of days >60 minutes PA in average week				
≥5 days	219 (51.4)	61 (50.0)	53 (33.1)	<0.001
**Weekend**	Low use^d^	Medium use	High use	
n = 717^a^	414 (57.7)	108 (15.1)	195 (27.2)	
Gender (female)	294 (71.0)	35 (32.4)	55 (28.2)	<0.001
Parental education (≥13y)	247 (62.5)	66 (62.9)	100 (53.8)	0.11
Ethnicity (minority)	38 (9.2)	8 (7.4)	17 (8.7)	0.85
Number of days >60 minutes PA in average week				
≥5 days	213 (52.5)	43 (40.2)	76 (39.6)	0.003

^a^Minor discrepancies in size from sample total exist owing to missing values.

^b^Low use: adolescents playing electronic games during weekday 0–1 hours; Medium use: 1.5–2 hours; High use: adolescents playing electronic games ≥2.5 hours (per day during weekdays).

^c^Chi-square test between groups categorized by hours spent playing electronic games during the weekday or weekend.

^d^Low use: adolescents playing electronic games during weekend 0–1.5 hours; Medium use: 2–2.5 hours; High use: adolescents playing electronic games ≥3 hours (per day during weekend days).

#### Weekend

The median time spent playing online games by the adolescents on weekend days was 1.5 hour. A total of 58% of adolescents played electronic games ≤1.5 hours, 15% between 2–2.5 hours, and 27% ≥3 hours per weekend. Analyses of the sample grouped by median-based categories for weekend gaming revealed the low use group having significantly higher proportions of females (Table 3). There was a significant association between PA and electronic game playing during weekend days (p = 0.003). The percentage of those reporting PA over 60 minutes ≥5 days was 52.5%, 40.2%, 39.6% among the low use, medium use, and high use groups respectively.

### Online socializing or surfing habits

#### Weekday

The median time spent on online socializing by the adolescents on weekdays was 1.5 hours. A total of 58% of adolescents socialized or surfed online ≤1.5 hours, 18% between 2–2.5 hours, and 24% ≥3 hours per weekday. When comparing socio-demographic characteristics of the sample grouped by median-based categories for weekday socializing and surfing online, significant associations were identified for gender and parent education, with the highest proportion of males and those with parents educated ≥13 years found amongst the low use group (Table 4). There was a significant association between PA and socializing or internet surfing during weekdays (p = 0.01). The percentage of those reporting PA over 60 minutes ≥5 days was 51.3%, 45.5%, 38.1% among the low use, medium use and high use groups respectively.

**Table 4:** Socio-demographic and physical activity (PA) characteristics for sample total (n = 742), grouped by low, medium and high online socializing or internet surfing for weekday and weekend.

**Socializing/surfing online**	Low use^b^	Medium use	High use	P value^c^
**Weekday**	n (%)	n (%)	n (%)	
n = 722^a^	418 (57.9)	133 (18.4)	171 (23.7)	
Gender (female)	206 (49.3)	74 (55.6)	107 (62.6)	0.01
Parental education (≥13y)	274 (68.3)	66 (51.6)	77 (47.5)	<0.001
Ethnicity (minority)	35 (8.4)	15 (11.3)	15 (8.8)	0.59
Number of days >60 minutes PA in average week				
≥5 days	211 (51.3)	60 (45.5)	64 (38.1)	0.01
**Weekend**	Low use^d^	Medium use	High use	
n = 720^a^	392 (54.4)	121 (16.8)	207 (28.8)	
Gender (female)	188 (48.0)	70 (57.9)	128 (61.8)	0.003
Parental education (≥13y)	261 (69.2)	56 (49.6)	101 (50.8)	<0.001
Ethnicity (minority)	33 (8.4)	11 (9.1)	21 (10.1)	0.78
Number of days >60 minutes PA in average week				
≥5 days	200 (51.8)	48 (39.7)	88 (43.1)	0.03

^a^Minor discrepancies in size from sample total exist owing to missing values.

^b^Low use: adolescents socializing/surfing online during weekday 0–1.5 hours; Medium use: 2–2.5 hours; High use: adolescents socializing/surfing online ≥3 hours (per day during weekdays).

^c^Chi-square test between groups categorized by hours spent socializing or surfing online during the weekday or weekend.

^d^Low use: adolescents socializing/surfing online 0–1.5 hours; Medium use: 2–2.5 hours; High use: adolescents socializing/surfing online ≥3 hours (per day during weekend days).

#### Weekend

The median time spent on online socializing by the adolescents on weekend days was 1.5 hours. A total of 54% of adolescents socialized or surfed online ≤1.5 hours, 17% between 2–2.5 hours, and 29% ≥3 hours per weekend. Analyses of the sample grouped by median-based categories for weekend socializing and surfing online revealed the low use group having significantly higher proportions of males and parents with an education ≥13 years (Table 4). There was a significant association between PA and socializing or internet surfing during weekend days (p = 0.03). The percentage of those reporting PA over 60 minutes ≥5 days was 51.8%, 39.7%, 43.1% among the low use, medium use and high use groups respectively.

### Regression analyses

Multiple logistic regression analyses for weekday screen-based SB revealed that the low use and medium use groups watching television/movie streaming during the weekdays had significantly higher odds of performing PA over 60 minutes ≥5 days in the average week compared to the high use group (aOR = 1.76, 95% CI 1.10–2.83, and aOR = 2.17, 95% CI 1.28–3.70, p = 0.01, respectively; Table 5). Regarding electronic games, the low use group had significantly higher odds of participating in PA over 60 minutes ≥5 days in the week than the high use group (aOR = 2.25, 95% CI 1.31–3.85, p = 0.01). There were no significant associations regarding PA and socializing/surfing online.

**Table 5:** Factors associated with participating in physical activity (PA) over 60 minutes ≥5 days in the average weekday.

		Crude		Adjusted	
	Group	cOR^a^ (95% CI)	P value	aOR (95% CI)	P value
TV watching^b^	High use^c^	1.00	<0.001	1.00	0.01
	Medium use	2.31 (1.48–3.60)		2.17 (1.28–3.70)	
	Low use	2.47 (1.68–3.62)		1.76 (1.10–2.83)	
Electronic gaming^d^	High use^e^	1.00	<0.001	1.00	0.01
	Medium use	2.02 (1.24–3.28)		1.51 (0.83–2.77)	
	Low use	2.14 (1.46–3.12)		2.25 (1.31–3.85)	
Socializing/surfing^b^	High use^f^	1.00	0.01	1.00	0.84
	Medium use	1.35 (0.85–2.15)		1.06 (0.60–1.85)	
	Low use	1.71 (1.19–2.47)		1.14 (0.72–1.81)	

^a^Crude and adjusted odds ratios (cOR/aOR).

^b^Adjusted for gender, ethnicity, parental education, self-efficacy for television watching, self-efficacy for PA, having a television in bedroom, owning own PC, number of PC’s in home, doing homework during the week, doing hobbies during the week.

^c^High use: adolescents watching TV during weekday ≥3 hours (per day during weekdays); Medium use: 2–2.5 hours; Low use: adolescents watching for 0–1.5 hours.

^d^Adjusted for the same elements as footnote b, with the addition of self-efficacy for electronic games.

^e^High use: adolescents playing electronic games ≥2.5 hours (per day during weekdays); Medium use: 1.5–2 hours; Low use: 0–1 hours.

^f^High use: adolescents socializing/surfing online ≥3 hours (per day during weekdays); Medium use: 2–2.5 hours; Low use: weekday 0–1.5 hours.

In the multiple logistic regression analyses regarding weekend screen-based SB, significantly higher odds for participating in PA over 60 minutes ≥5 days in the week were found for those watching television/movie streaming in the medium use group (aOR = 2.29, 95% CI 1.27–4.13, p = 0.02; Table 6) compared to the high use group. Regarding electronic games, the low use group had significantly higher odds of participating in PA over 60 minutes ≥5 days in the average week compared to the high use group (aOR = 1.83, 95% CI 1.11–3.03, p<0.001). By contrast, the medium use group had significantly lower odds of participating in PA over 60 minutes ≥5 days in the average week compared to the high use group (aOR = 0.52, 95% CI 0.28–0.96, p<0.001). There were no significant associations regarding PA and socializing/surfing online.

**Table 6:** Factors associated with participation in physical activity (PA) over 60 minutes ≥5 days in the week during the average weekend.

		Crude		Adjusted	
	Group	cOR^a^ (95% CI)	P value	aOR (95% CI)	P value
TV watching^b^	High use^c^	1.00	<0.001	1.00	0.02
	Medium use	2.65 (1.61–4.34)		2.29 (1.27–4.13)	
	Low use	2.54 (1.63–3.95)		1.69 (0.98–2.91)	
Electronic gaming^d^	High use^e^	1.00	0.004	1.00	<0.001
	Medium use	1.03 (0.63–1.66)		0.52 (0.28–0.96)	
	Low use	1.68 (1.19–2.39)		1.83 (1.11–3.03)	
Socializing/surfing^b^	High use^f^	1.00	0.03	1.00	0.23
	Medium use	0.87 (0.55–1.37)		0.61 (0.34–1.07)	
	Low use	1.42 (1.01–1.99)		0.85 (0.55–1.31)	

^a^Crude and adjusted odds ratios (cOR/aOR).

^b^Adjusted for gender, ethnicity, parental education, self-efficacy for television watching, self-efficacy for PA, having a television in bedroom, owning own PC, number of PC’s in home, doing homework during the weekend, doing hobbies during the weekend.

^c^High use: adolescents watching ≥4 hours (per day during weekend days); Medium use: 3–3.5 hours; Low use: 0–2.5 hours.

^d^Adjusted for the same elements as footnote b, with the addition of self-efficacy for electronic games.

^e^High use: adolescents playing electronic games ≥3 hours (per day during weekend days); Medium use: 2–2.5 hours; Low use: 0–1.5 hours.

^f^High use: adolescents socializing/surfing online ≥3 hours (per day during weekend days); Medium use: 2–2.5 hours; Low use: 0–1.5 hours.

## Discussion

The study aimed to assess whether time spent performing three different screen-based SBs was associated with time spent participating in PA among adolescents. The results showed that those with low (vs high) time spent on TV/movie streaming and electronic game playing had significantly higher odds of participating in physical activity (PA) over 60 minutes ≥5 days during weekdays and weekend days. Of the three screen-based activities, electronic gaming ≤1 hour per day for weekdays and TV viewing between 3–3.5 hours per day for weekend days resulted in the highest significant odds for participating in PA over 60 minutes. There were no significant associations between socializing/surfing online both on weekdays and weekend days and physical activity in adjusted models.

The results of this study serve to illustrate the impact different screen-based SBs have upon young adolescents’ ability to participate in enough PA to satisfy the minimum amount recommended in national and international health guidelines, such as those outlined by the World Health Organisation [27]. Adolescents with parents having low education levels were significantly more likely to have a higher screen time. In their review of sedentary behaviour in youth, Pate et al. [12] report that children from low socio-economic households spent more time in screen-based SBs, irrespective of the indicator of socioeconomic status used (parental education, income, employment). Another Norwegian study also reported that a lower level of parent education resulted in a significantly higher proportion with a screen-time ≥2 hours/day [28], further supporting our findings here.

Previous studies have explored the association between screen-based SB, in particular TV/movie streaming and electronic games, and time spent on PA, and documented inverse associations [14, 29, 30]. This is mostly in line with findings of this study, although the inverse association was not altogether consistent when stratified median-based exposure times of screen-based SBs were used, as reported in our regression results. While both low and medium weekday and weekend day TV user groups both had higher odds for PA compared to the high weekday and weekend day TV use group, the medium TV use group had somewhat higher odds than the low TV use group. Likewise, the weekend medium electronic game use group had lower odds for PA compared to the high user group as well as the low use group. These anomalies might be owing to our use of median value cut-offs to delineate between low, medium and high groups for the various SB’s, or else may highlight a novel characteristic regarding moderate screen-based SB users when compared to low or high use groups.

In the present study, we also explored whether online socializing or surfing habits had a similar effect upon PA as the other screen-based SBs. Although our regression results supported studies regarding the impact high levels of TV/movie streaming and electronic gaming have on PA [14, 29–31], we found no significant association between socializing/surfing online and PA, either for weekday or weekend days. These differences in the associations between PA and the different SBs included are in line with suggestions by Biddle and colleagues that different screen-based SB behaviours should be considered as separate behaviours [32]. The null associations between online socializing/surfing habits and PA found in the present study are in line with two other studies among adolescents [32, 33]. By contrast, a Dutch study among adolescents reported that >2 hours/day internet use significantly affected adolescents’ ability to participate in PA ≥60 minutes/day [29]. The contrasting results may be due, at least in part, to different cut-offs used to define high sedentary time, an inability of respondents to distinguish clear boundaries between different screen-based behaviours, and differing measures used for assessing PA and SB. The lack of an association between online socializing/surfing habits and PA in the regression analysis, might also be explained by the immediate and extensive accessibility of online activity to adolescents throughout the day. This ‘all day online accessibility’ would be possible via mobile smartphone use, and children aged 11 and older have previously been reported as having ubiquitous access to such devices [34]. This may create a scenario making an unaffected PA time allocation for adolescents plausible, although a moderate inverse association between smartphone use and PA has previously been shown in a similar population [35].

There were no differences in the associations found in this study between weekdays and weekend days, which did not support the hypothesis that weekend activities displaced time available in a different way for either SB or PA compared to weekdays [20]. The displacement theory is no stranger to debate, as it has been somewhat inconsistent in the literature with the implication that PA and SBs tend to replace each other [36–39]. However, evaluation of displacement also requires that other factors such as light PA and sleep are also taken into consideration, thus no firm conclusions can be drawn in this regard.

### Strengths and limitations

This study should be interpreted within the context of important strengths and weaknesses. A large sample size with a high response rate at the school level and moderate response rate at the parental level provided robust data. The inclusion of different SBs and separate weekday and weekend assessments of SB was another strength of the study. That this study is based upon cross-sectional data, thereby limiting our ability to determine any causal inference may be considered a weakness of this study. Whilst some of the literature assesses PA data based upon objective instrumentation such as heart rate monitors or activity monitors, our assessment of PA levels is based upon self-reported data. Although no measurement practise is without limitations, a number of studies have reported differences in PA levels when self-reported data were compared with objective methods [40–42]. Limitations regarding the assessment of SB’s amongst adolescents have also been previously reported [43]. However, the measures of SB and PA used in this study have previously shown evidence of reliability or validity among a similar population [23–26]. Although several confounders were adjusted for in the analyses, factors such as body weight were not measured and were thus not adjusted for. Finally, the study is limited to a single geographical area, making generalizability limited.

## Conclusion

TV/movie streaming and electronic game playing during both weekdays and weekend days were significantly inversely related with participating in physical activity. Initiatives aimed at reducing screen-based sedentary activities might result in favourable effects on physical activity levels among adolescents.

## Supporting information

S1 File (DOCX)
