# Supplementary material for: Comparing three screen-based sedentary behaviours’ effect upon adolescents’ participation in physical activity: The ESSENS study
Source: PLoS One. 2020 Nov 25;15(11):e0241887. doi: 10.1371/journal.pone.0241887 (PMC7688164; doi:10.1371/journal.pone.0241887)
Supplement: S1 File — (DOCX) [file pone.0241887.s001.docx]

**Are you a boy or a girl?**

 Girl

 Boy

**In which country was your mother born?**

 Norway

 Other country

**In which country was your father born?**

 Norway

 Other country

**How many hours do you usually watch TV as well as DVD, video or films on a PC, telephone or Ipad in your free time?**

**Tick off one box for weekdays and one for weekend days**

**Weekdays (average of all weekdays)**

 None

 Less than 30 minutes per day

 1,0 hour/day

 1,5 hours/day

 2,0 hours/day

 2,5 hours/day

 3,0 hours/day

 3,5 hours/day

 4,0 hours/day or more

**Weekend day (average of a Saturday or a Sunday)**

 None

 Less than 30 minutes per day

 1,0 hour/day

 1,5 hours/day

 2,0 hours/day

 2,5 hours/day

 3,0 hours/day

 3,5 hours/day

 4,0 hours/day or more

**How many hours per day do you usually play computer games or electronic games (Playstation, XBOX etc), or play games on an iPad/tablet or mobile phone?**

**Weekdays (average of all weekdays)**

 None

 Less than 30 minutes per day

 1,0 hour/day

 1,5 hours/day

 2,0 hours/day

 2,5 hours/day

 3,0 hours/day

 3,5 hours/day

 4,0 hours/day or more

**Weekend day (average of a Saturday or a Sunday)**

 None

 Less than 30 minutes per day

 1,0 hour/day

 1,5 hours/day

 2,0 hours/day

 2,5 hours/day

 3,0 hours/day

 3,5 hours/day

 4,0 hours/day or more

**How many hours per day do you usually use a computer, iPad/tablet or mobile phone for activities such as chatting, e-mails, internet surfing, Facebook or Instagram in your free time?**

**Tick off one box for weekdays and one for weekend days**

**Weekdays (average of all weekdays)**

 None

 Less than 30 minutes per day

 1,0 hour/day

 1,5 hours/day

 2,0 hours/day

 2,5 hours/day

 3,0 hours/day

 3,5 hours/day

 4,0 hours/day or more

**Weekend day (average of a Saturday or a Sunday)**

 None

 Less than 30 minutes per day

 1,0 hour/day

 1,5 hours/day

 2,0 hours/day

 2,5 hours/day

 3,0 hours/day

 3,5 hours/day

 4,0 hours/day or more

**Do you have a TV in your bedroom?**

Yes

No

**Do you have your own computer?**

Yes

No

**Do you have your own ipad?**

Yes

No

**How sure are you that you can…. (answer categories: sure/not sure)**

**When we say TV viewing we also mean watching DVD, video or films on a PC, telephone or Ipad**

Limit TV viewing to 1 hour at least one school day

Limit watching TV to 1 hour per day for most schools days

Limit TV viewing to 1 hour at least one non-school day, including the weekend

Not watch TV at all for most school days

Not watch TV at all for most non-school days, including weekend

**How sure are you that you can…. (answer categories: sure/not sure)**

Limit computer games, game on consoles, games on ipad or on phone to1 hour at least one school day?

Limit computer games, game on consoles, games on ipad or on phone to 1 hour per day on most school days?

Limit computer games, game on consoles, games on ipad or on phone to 1 hour at least one non-school day, including the weekend

Not play computer games, game on consoles, games on ipad or on phone at all for most school days

Limit computer games, game on consoles, games on ipad or on phone at all for most non-school days, including weekend

**How many hours do you use on the following activities on a weekday?**

**How many hours do you use to do homework or read a book?**

Less than half hour

Half an hour

1 hour

1,5 hours

2 hours

2,5 hours

3 hours

More than 3 hours

**How many hours do you use for hobbies, handcrafts or music lessons/practise?**

Less than half hour

Half an hour

1 hour

1,5 hours

2 hours

2,5 hours

3 hours

More than 3 hours

**How many hours do you use on the following activities on a weekend day?**

**How many hours do you use doing homework or reading a book?**

Less than half hour

Half an hour

1 hour

1,5 hours

2 hours

2,5 hours

3 hours

More than 3 hours

**How many hours do you use doing hobbies, handcrafts or music lessons/practise?**

Less than half hour

Half an hour

1 hour

1,5 hours

2 hours

2,5 hours

3 hours

More than 3 hours

**During the course of a typical or regular week, on how many are you usually active for at least 60 minutes per day?**

**Physical activity includes doing training, playing and engaging in sports**

0 days 1 day 2 days 3 days 4 days 5 days 6 days 7 days

**To what extent do you agree with the following statements (completely disagree to completely agree response options) ?**

I manage to be physically active on most days

I manage to ask my parents or other adults to train, play or engage in physical activity with me

I manage to be physically active on most days even when I have the opportunity to watch TV or play electronic games instead

I manage to be physically active on most days even though the weather is bad outside

I manage to bring along my friends for physical activity on most days
